# Receptor-Targeted, Magneto-Mechanical Stimulation of Osteogenic Differentiation of Human Bone Marrow-Derived Mesenchymal Stem Cells

**DOI:** 10.3390/ijms140919276

**Published:** 2013-09-23

**Authors:** Bin Hu, Alicia J El Haj, Jon Dobson

**Affiliations:** 1 Institute of Science and Technology in Medicine, Guy Hilton Research Center, Keele University, Thornburrow Drive, Hartshill, Stoke on Trent, ST4 7QB, UK; E-Mails: b.hu@keele.ac.uk (B.H.); a.j.el.haj@keele.ac.uk (A.J.E.H.); 2 J. Crayton Puritt Family Department of Biomedical Engineering, Department of Materials Science and Engineering, Institute of Cell Engineering and Regenerative Medicine (ICERM), University of Florida, Gainesville, FL 32611, USA

**Keywords:** magnetic force bioreactor, tissue engineering, mechanotransduction, human mesenchymal stem cells, osteogenic differentiation

## Abstract

Mechanical cues are employed to promote stem cell differentiation and functional tissue formation in tissue engineering and regenerative medicine. We have developed a Magnetic Force Bioreactor (MFB) that delivers highly targeted local forces to cells at a pico-newton level, utilizing magnetic micro- and nano-particles to target cell surface receptors. In this study, we investigated the effects of magnetically targeting and actuating specific two mechanical-sensitive cell membrane receptors—platelet-derived growth factor receptor α (PDGFRα) and integrin α_ν_β_3_. It was found that a higher mineral-to-matrix ratio was obtained after three weeks of magneto-mechanical stimulation coupled with osteogenic medium culture by initially targeting PDGFRα compared with targeting integrin α_ν_β_3_ and non-treated controls. Moreover, different initiation sites caused a differentiated response profile when using a 2-day-lagged magneto-mechanical stimulation over culture periods of 7 and 12 days). However, both resulted in statistically higher osteogenic marker genes expression compared with immediate magneto-mechanical stimulation. These results provide insights into important parameters for designing appropriate protocols for *ex vivo* induced bone formation via magneto-mechanical actuation.

## 1. Introduction

Cell-based bone tissue engineering is one essential strategy to treat and repair bone defects resulting from disease or traumatic injury. Traditionally, auto- or allo- bone grafts have been used to repair damaged bone. However, this modality is limited by factors such as limited availability, donor site morbidity and host immune rejection. Cell-based bone tissue engineering exploits *in vitro* expanded progenitor or stem cells, biodegradable scaffolds and other stimulatory biochemical or mechanical factors to induce the appropriate differentiation of cells to create implants for a defect site [1–3]. The proliferation capacity and differentiation potential are among the critical considerations for the success of the strategy. Although embryonic stem (ES) cells have ignited interest recently in their potential for clinical applications, due to the difficulties in obtaining homogenous populations of differentiated cells, the risk of teratoma formation, and complex ethical issues, ES cells have not been widely adopted in the clinic to date in most countries. Human bone marrow-derived mesenchymal stem cells (hMSCs), on the other hand, while more limited in their differentiation potential, are able to form clinically relevant tissues.

In addition, they are relatively widely available, somewhat immuno-inert, and have been widely used for clinical trials for a variety of diseases including bone tissue defects as a consequence. In addition, mechanical stimulation has been proposed for pre-conditioning of osteoblast and stem cell-seeded constructs, ultimately for the production of tissue engineered bone with enhanced differentiation, mineralization and mechanical properties [4]. As such, not only the quality of tissue-engineered products but also the time period necessary for the generation of *ex vivo* bone could potentially be improved using mechanical stimulation. Techniques applying compressive or tensile loading to mechanically precondition cell-seeded constructs *in vitro* prior to implantation have been developed [5]. However, these approaches rely heavily on the mechanical properties of the biodegradable scaffolds used. There is an unmet need in tissue engineering applications for the development of techniques that apply forces directly to cells or even to mechano-responsive receptors within individual cells, bypassing the need to deform the surrounding scaffold as a whole and thereby rendering moot the requirement for mechanically strong scaffolds [5].

**Figure 1:**
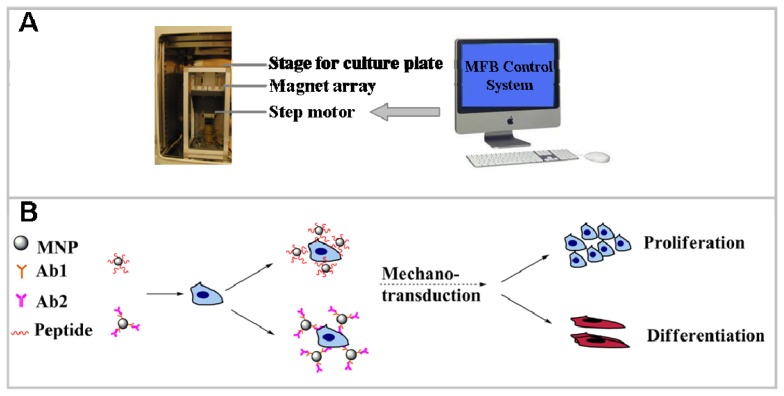
Schematic diagram (**A**) and proposed underlying mechanism (**B**) of Magnetic Force Bioreactor (MFB).

The Magnetic Force Bioreactor (MFB) technique is a novel method of applying mechanical forces, in the pico-newton (pN) and nano-newton (nN) range, directly to molecular components of cells mediated by functionalized micro- or nano-particles over varying incubation periods for different purposes (Figure 1). While there are many studies focusing on the biological effects of magnetic fields, conflicting results have been demonstrated [6,7]. The MFB technique used in this study, combining magnetic fields with magnetic nanoparticles (MNPs) targeting specific cell surface receptors to control specific cell signaling pathways, is based on magneto-mechanical transduction. In this case, MNPs are the local ‘force generators’ transducing an applied magnetic field to elicit a conformational change in a membrane protein/receptor in order to activate a specific signaling pathway. Furthermore, the incorporation of MNPs expands the potential application field of magnetic tissue engineering techniques as MNPs functionality can be tailored by controlling MNP properties such as size, surface chemistry and bio-functionalization [8–12]. As such, targeting and activating diversified signaling transduction pathways and ensuing cell lineage commitment or tissue formation may potentially be achieved.

In our previous investigations, as well as in results reported here, field only controls were incorporated into the experiment design. In all cases, no apparent biological effects have been observed solely by application of magnetic field on cells. Iron oxide MNPs have been used as magnetic resonance imaging (MRI) contrast agent in the clinic and for cell isolation in clinical trials, and their biocompatibility is well documented as several iron-oxide/polymer composites have been cleared for clinical use by the U.S. Food and Drug Administration (FDA).

Previous work has shown that the MFB can be utilized to apply mechanical forces to control cells for cell and tissue engineering applications [13–16]. Though these preliminary studies show potential, several questions relate to potential oncogenic effects, thorough toxicology studies, integrity and durability of the MFB-generated tissue matrix, and effects of time and sequence on activation of multiple receptors. However, magneto-mechanical activation of cell signaling pathways still has great potential to allow the usage of a variety of non-load-bearing matrices/scaffolds and also is capable of augmenting the mechanical strength and reducing the *in vitro* preparation time of bone tissue engineered constructs. Magneto-mechanical activation of MNP-tagged specific mechanosensitive receptors, TREK-1 and integrin, can induce adult hMSCs to differentiate along the osteochondrogenic lineage [17]. This technology represents an intriguing modality for long-term bone tissue engineering study. However, the spatio-temporal relationship between biochemical signaling activation and mechanical cues to promote differentiation is not yet clear [18–21].

Platelet-derived growth factor (PDGF) and PDGF receptors (PDGFRs) are also implicated in osteogeneis and bone formation, although their influence remains controversial. It is generally accepted that PDGF is not the sole factor capable of inducing osteogenic differentiation of MSCs [22]. PDGF ligands and receptors are elevated during tissue remodelling in bone fractures. *In vivo* application of PDGF has been shown to enhance bone formation in some reports [23–25] but confer inhibitory effects in other cases [26]. Imatinib-induced blockade of PDGFR(β) showed suppressive effects on the osteogenic differentiation of MSCs [27]; whereas promoting effects on the differentiation of osteoblasts and bone formation have been demonstrated in several reports [28–30]. In our previous study, short-term mechanical stimulation of PDGFRs via MFB induced myogenesis of hMSCs [14]. However, no study of direct mechanical stimulation of PDGFR combined with osteogenic biochemical factors has been reported so far.

In this study, we aim to define and characterize the protocols and parameters which are required for controlling hMSCs differentiation *in vitro* for bone tissue engineering. We describe optimization studies demonstrating the effects of magnetic particle presence and force application on osteogenesis and mineralization. The effect of initial targeting and mechanical stimulation of mechanosensitive platelet-derived growth factor receptor α (PDGFRα) and integrin over a long period, combined with osteogenic supplements on the osteogenesis of hMSCs, is examined. The temporal effects of mechanical stimuli at different stages of the osteogenic differentiation of hMSCs to promote maximal response are also explored. It is hypothesized that magnetic particle based techniques represent a valuable tool for stimulating and remotely controlling the differentiation of stem cells to osteoblasts over prolonged periods of time, and have potential applications in tissue engineering and regenerative medicine.

## 2. Results and Discussion

### 2.1. Magneto-Mechanical Stimulation Coupled with Biochemical Factors Directs the Osteogenic Differentiation of hMSCs

**Figure 2:**
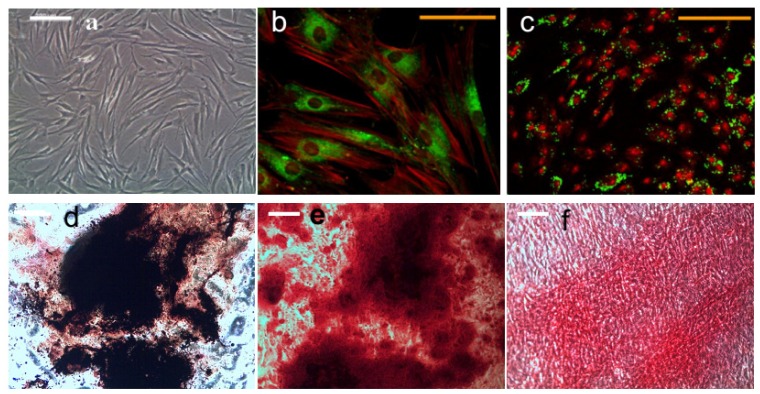
Morphology of undifferentiated hMSCs (**a**); magnetic nanoparticle (MNP) targeting PDGFRα (**b**) and integrin α_ν_β_3_ (**c**) in hMSCs, and histology analyses of hMSCs after 21 days of osteogenic induction (**b**–**d**). (**a**) Light microscope image, scale bar = 200 μm; (**b**) Confocal image of dextran (green) and F-actin (red) staining showing PDGFRα antibody functionalized MNP targeting in hMSCs, scale bar = 100 μm; (**c**) Confocal image of dextran (green) and nucleus (red) staining showing RGD peptide functionalized MNP targeting in hMSCs; (**d**) Alkaline phosphatase and von Kossa staining; (**e**) Alizarin red s staining; (**f**) Sirius-red staining. (**c**–**f**) Scale bar = 200 μm.

HMSCs were plated onto 6-well plates and cultured in growth medium to confluence. After 3 weeks of daily application of magneto-mechanical stimulation, no mineral deposits were observed in groups without osteogenic supplements, whilst regions containing thickened fibers and large mineral deposits were observed when osteogenic supplements were added. HMSCs treated with magneto-mechanical stimulation combined with biochemical factors form mineralized nodules, which stain positively for calcium and phosphorous as demonstrated using co-staining of Alizarin Red S and von Kossa (Figure 2). These stains may have been localized to regions of dystrophic mineralization.

**Figure 3:**
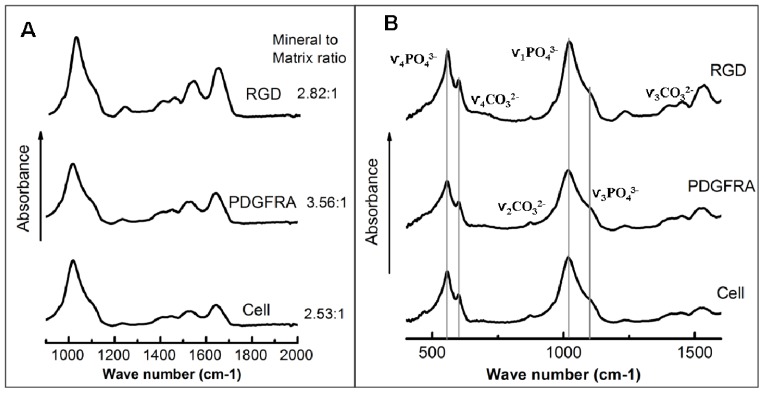
FTIR spectra obtained before (**A**) and after (**B**) thermal gravimetric analysis of mechanical stimulated hMSCs (PDGFRα and RGD treatment) and non-treated hMSCs (Cell) following 3 weeks of culture. In both the PDGFRα treatment (denoted PGFRA in the plot) and RGD treatment (denoted RGD in the plot) groups, hMSCs were incubated with the appropriate antibody-conjugated MNPs and exposed to the MFB for 1 h per day for 3 weeks. The MFB exposure frequency was set as 1 Hz. Medium was changed every other day. The mineral-to-matrix ratio was obtained by integrating the area under the curve between 900 and 1200 cm^−1^ (ν_1_, ν_3_ PO_4_ band) and dividing by the area under the curve between 1585 and 1725 cm^−1^ (amide I band) [32].

FTIR analyses were conducted in order to examine and compare the mineralized ECM of hMSCs (Figure 3) and rule out the possibility of dystrophic mineralization. FTIR spectra corresponding to the 3 sample types are displayed in Figure 3 for the wavenumber range of 400–1600 cm^−1^. This region covers four important components, *i.e.*, the ν_4_ PO_4_^3−^ region (500–650 cm^−1^), which reveals apatitic and non-apatitic HPO_4_^2−^ or PO_4_^3−^ environments, the ν_1_ and ν_3_ phosphate (910–1180 cm^−1^), and the ν_2_ carbonate band region (approximately 900–910 cm^−1^). The mineral-to-matrix ratio, crystallinity, and collagen cross-link ratio (XLR) of different groups are summarized in Table 1. The spectra obtained from the samples were evaluated as being carbonated hydroxyapatite minerals with variable carbonate substitutions for phosphate. The sub-bands at 1125 and 1020 cm^−1^ are characteristic of an immature hydroxyapatite rich in acid phosphate and carbonate substituents. However, human bone has a mineral-to-matrix ratio of 4.7:1 [31], which was substantially higher than for the ECM produced by hMSCs receiving different treatments (3.6:1, PDGFR-MNP; 2.8:1, RGD-MNP; 2.5:1, non-treated cells) in the presence of osteogenic media supplements. For 3 weeks magneto-mechanical stimulated groups, the mineral-to-matrix ratio of PDGFRα treatment group was significantly upregulated compared with non-treatment group assessed by FTIR (Table 1).

**Figure 4:**
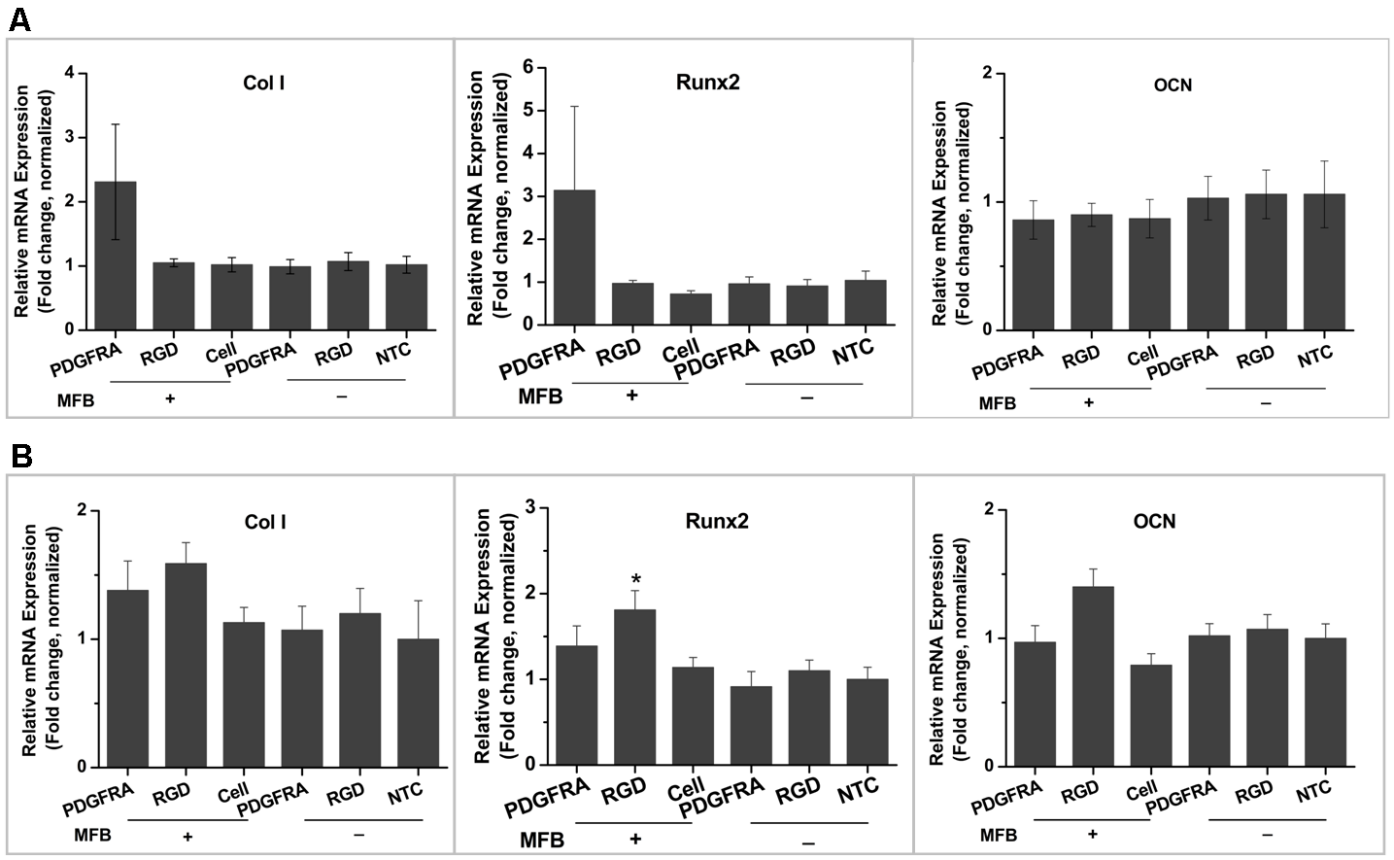
Osteogenic marker gene expression (relative fold change normalized to each control group) in hMSCs following 3 weeks of exposure to the MFB with cells cultured in (**A**) proliferation medium and (**B**) osteogenic medium (*n* = 3, mean ± SD, ^*^p < 0.05). Experimental and control groups: PDGFRA: cells treated with PDGFRα antibody-conjugated MNPs; RGD: cells treated with RGD-conjugated MNPs; Cells: cells only (with field); NTC: non-treated control; MFB “+”: exposed to magnetic fields; MFB “−”: no field controls.

Osteogenic marker expression was analysed over 3 weeks of culture in either proliferation medium or osteogenic medium (Figure 4). It is observed that collagen type I (Col I) and Runx 2 were enhanced in experiment groups receiving MFB treatment via RGD- or PDGFRα antibody- conjugated MNPs cultured in proliferation medium, although detected changes were not statistically significant compared with the non-treated control group. Whereas in osteogenic medium cultured cells, magnetic conditioning via PDGFRα antibody- functionalized MNPs, RGD-functionalized MNPs and MNP without conjugation exhibited enhanced Col I and Runx2 expression, while RGD-conjugation caused significant changes compared with the non-treated group. It is worth noting that Figure 4A,B shows the relative mRNA expression in different samples, and the Δ*Ct* values of three target genes are much smaller in samples cultured in osteogenic medium compared with those cultured in proliferation medium (data not shown). This indicates that the established osteogenic differentiation of hMSCs in osteogenic medium cultured groups, which is consistent with the aforementioned histology staining and FTIR spectra data. In addition, the observed high errors in the group treated by PDGFRα antibody-conjugated MNPS combined with MFB exposure may be caused by the low expression level of PDGFRα in hMSCs compared with integrin, which led to varied targeting efficiency across different samples when cultured in proliferation medium; whereas when cell were cultured in osteogenic medium, the variation of targeting efficiency possibly was masked by the biochemical cue-induced osteogenesis.

### 2.2. PDGFR and Osteogenesis of hMSCs

Osteoblasts arise from mesenchymal stem cells and the transition from stem cells to mature osteoblast is characterized by the formation of mineralized extracellular matrix and controlled by key osteogenic transcription factors, such as Runx2 and osterix [35]. The interplay of hormones, growth factors, and mechanical cues directs the osteogenic differentiation of hMSCs *in vivo*. The complexity of temporally and spatially orchestrating various factors for bone formation hinders the development of engineered functional bone. Bone morphological factors (BMPs) are one type of growth factors with wide application in research and clinical. PDGF also attracts emerging research efforts for its potential role in bone formation; however, effect of PDGF on osteogenesis of hMSCs is still in debate [36]. Inconsistent results may have resulted from differences in experimental conditions, especially the difference between *in vitro* and *in vivo*. However, evidence has shown that PDGF signaling is not competent to act as a sole factor in directing the commitment of hMSCs to the osteoblasts [22]. Results in this study confirmed that activation of PDGFRα alone is not sufficient to drive the differentiation of hMSCs down the osteoblast lineage in the absence of osteogenic supplements.

**Figure 5:**
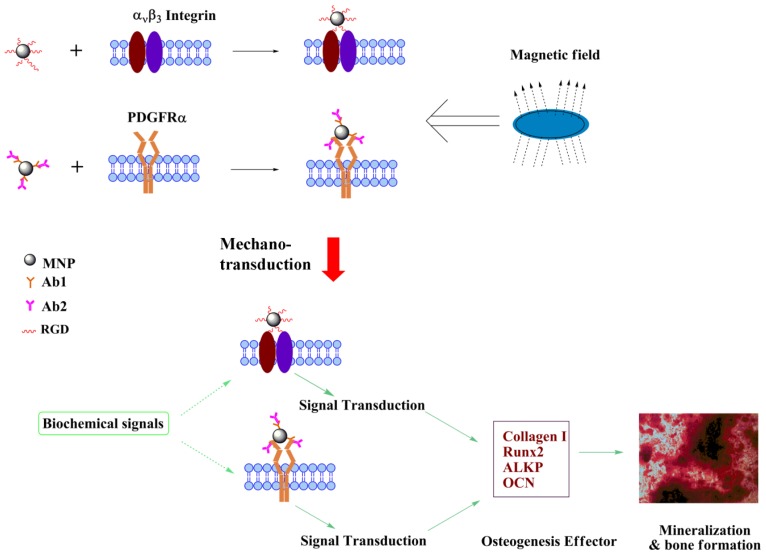
Schematic illustration of the proposed osteogenic differentiation of hMSCs induced by magneto-mechanical stimulation combined with biochemical signals. MNPs were functionalized with RGD peptide or PDGFRα antibody to target either integrin α_ν_β_3_ or PDGFRα on cell surface membrane of hMSCs. When exposed to MFB, induced mechano-transduction coupled with biochemical signals directed the osteogenic differentiation and mineralization of hMSCs.

In contrast, conditioning hMSCs using PDGFRα antibody-conjugated MNPs and the MFB enhanced the ECM mineralization after 3 weeks of culture in the presence of osteogenic supplements, and RGD-conjugated MNPs combined with MFB exposure augmented the mineral to matrix ratio to a lesser degree assessed by FTIR spectra (Figure 3). Cells treated with RGD-conjugated MNPs combined with MFB exposure showed enhanced expression of Col I, Runx2, ALKP and OCN, but significant changes were found only for Runx2. When considering that the expression levels of osteogenic markers are changed after magnetic conditioning, we hypothesize that: (1) Mechanical stimulation has a synergistic osteogenic function with the osteogenic medium; and (2) Following 3 weeks of mechanical stimulation, the MNPs may act as local force generators in the ECM and/or intracellular cytoplasm, possibly acting on intracellular force transducer components such as actin filaments. This might explain the increases in osteogenic marker expression in PDGFRα and integrin targeting combined with the MFB exposure group. A proposed schematic diagram outlining the mechanisms of magneto-mechanical stimulation-induced osteogenesis of hMSCs is illustrated in Figure 5. As reported by Thomas *et al.* [18], hMSCs responded to mechanical stimulation after they had advanced beyond a differentiation threshold, which may explain our observations in the proliferation medium cultured groups. Direct mechanical stimulation from multipotent hMSCs might not guide cells along the osteoblast/osteocytes differentiation pathway, which requires proper temporal starting point to show a response.

The actual scenarios and proportions of receptors occupied by MNPs remain to be elucidated. They may undergo similar processes to the trafficking and cycling of cell surface receptors upon cognate ligands engagement. Multiple studies have reported observations of receptor-mediated endocytosis of MNPs, which could provide anecdotal evidence for the fate of cell surface receptors occupied by MNPs. Diverse types of cell surface receptors and properties of MNPs are among the contribution factors for the scenarios ensuing the engagement of receptors by MNPs. It has been proposed that MNPs can be endocytosed together with targeted cell surface receptors or released from the receptors. It has been also been suggested that endocytosed receptors may maintain or is a necessary step to initiate their endogenous functionality following their endocytosis [37]. Moreover, considering the size of MNPs employed in our experiments, there may be a risk of masking endogenous ligand-induced receptor activation or reducing the targeting efficiency of MNPs. Taken together, we do not clearly understand the influence of the occupation of receptors by MNPs on the endogenous trafficking and recycling of receptors themselves. Further research is warranted in this area.

### 2.3. Proposed Effect of Combinational Temporal Factors and Targeting Sites of Magneto-Mechanical Stimulation on Osteogenic Induction of hMSCs

**Figure 6:**
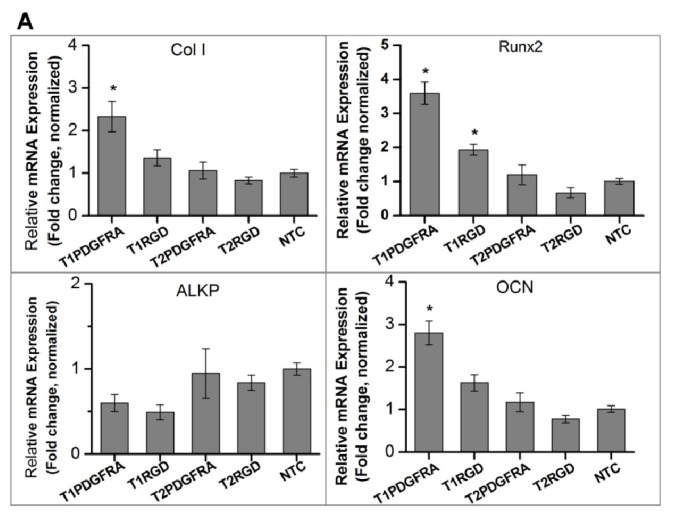

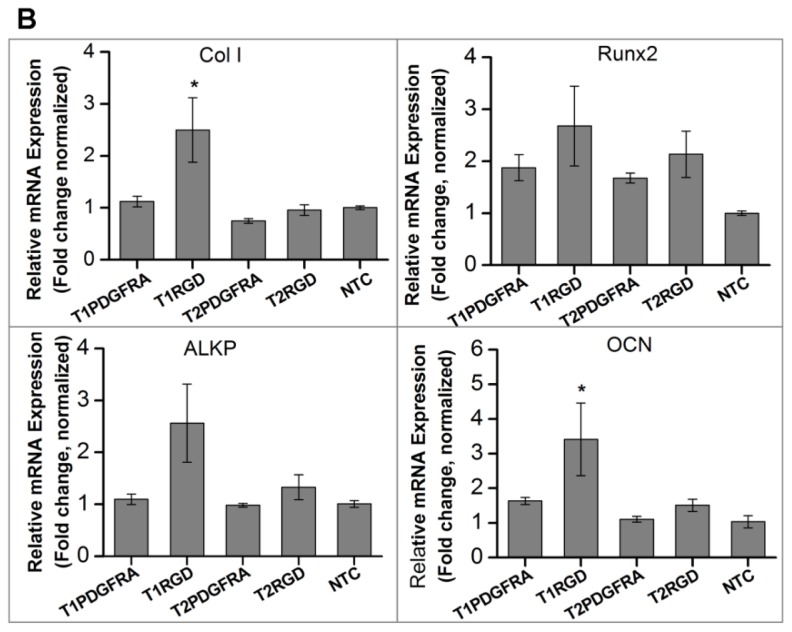
Effect of the temporal factor and targeting site of magneto-mechanical stimulation on osteogenic differentiation of hMSCs. Osteogenic marker gene expression, assessed by qRT-PCR, after 7 days of culture (**A**) and 12 days of culture (**B**). The T1 groups were preconditioned for 2 days by culturing in osteogenic medium under static conditions, followed by 5 days (**A**) or 10 days (**B**) of magnetic conditioning; The T2 groups were not preconditioned and instead underwent 7 days (**A**) or 12 days (**B**) of magnetic conditioning synchronized with osteogenic medium induction.

The influence of temporal and targeting sites of mechanical stimulation via PDGFRα- or RGD-conjugated MNPs on osteogenesis in hMSCs was investigated over a 7-day and 12-day period. For the temporal factors, the effects of preconditioning the cells by first culturing for 2 days in osteogenic induction medium were assessed. The groups of hMSCs that were and were not preconditioned with osteogenic medium are denoted T1 and T2 respectively. Firstly, as shown in Figure 6A, the levels of Runx2, Col I and OCN expression were upregulated in the group with 2 days of static osteogenic chemical induction (T1) compared to the group without static chemical preconditioning (T2) and non-treated (Control) group after 7 days of culture (Figure 6A). In contrast, the levels of alkaline phosphatase (ALKP) gene expression were lower in T1 with preconditioning compared to that in T2, the group without previous static osteogenic induction after 7 days of culture, and the control. However, the osteogenic marker gene expression profile was different after 12 days of culture compared to 7 days of culture: (i) RGD-conjugated MNPs combined with MFB exposure in T1 group induced Col I, ALKP and OCN expression compared with T2 groups, with Col I and OCN showing statistic significance; (ii) PDGFRα-conjugated MNPs combined with MFB appears to induce Runx2 and OCN expression in T1 groups compared with T2 groups, although no statistic significance was detected (Figure 6B). Osteogenic markers expression in mRNA level in T1 groups had no significant difference compared with non-treated control group after 7 days and 12 days post-culture. These results demonstrate that cells at various stages of differentiation have different sensitivities to mechanical conditioning. In the case of the markers for early differentiation, expression was enhanced with preconditioning using well defined chemical induction followed by mechanical stimulation. For early differentiation markers such as Runx2, and key matrix markers such as Col I and OCN, expression was enhanced with preconditioning. ALKP analyses demonstrated a different pattern; no statistic significance of gene expression was detected in T1 groups compared with that in T2 groups and the control group over 7 days or 12 days of culture. The asynchronous gene expression between ALKP and other osteogenic markers Col I, Runx2 and OCN possibly reflected that a peak expression of ALKP has been reached in one earlier time points, which have been demonstrated by other researchers [38,39]. The results also provide the insight into the role of magnetic mechano-conditioning in defining stem cell differentiation pathways and optimizing the temporal induction towards terminal osteogenesis.

We hypothesize that the MFB-based mechanical stimulation functions through the target-site specific and non-specific activation of downstream signaling pathways. For the target-site specific activation, the repertoire of cell surface molecules is critical for their interaction with antibody- or peptide- tagged MNPs, whereas for generic mechanical activation, the profile of extracellular matrix and intercellular cytoskeleton and other scaffold proteins decide the effect of mechanotranduction. It is conceivable that cells at different stages show differential responses to mechanical stimulation, including magneto-mechanical stimulation via MFB. Therefore, it is of great importance to explore the appropriately spatial and temporal factors for conditioning of hMSCs in order to augment the therapeutic effect of bone tissue engineering.

In this study, a short term (7 days) *in vitro* mechanical stimulation mediated by PDGFRα antibody-conjugated MNPs with 2-day pre-static culture in the presence of osteogenic supplements showed a better osteogenic induction effect *vs.* without 2-days pre-static culture, mediated by RGD-conjugated MNPs and the control. The difference in gene response of the hMSCs cells with no conditioning (without osteogenic medium induction) and 2-day conditioning can be attributed to osteogenic differentiation of hMSCs. The subsequent positive response to mechanical stimulation after pre-differentiation may be ascribed to an increase in cell surface receptors such as mechanosensitive integrins or the presence of ECM molecules to facilitate the physical connections between the mechanical stimuli and the cell [40]. ECM is important in the mechanotransduction process as it is crucial to interpret forces from the surrounding environment and to transmit these forces to the cell surface receptors (integrins) and then to the cytoskeleton (scaffolding proteins) [41,42]. The change in the mechanoresponsive behaviour of the hMSCs following pre-conditioning with osteogenic supplements can lead to diversified gene responses, which are dependent on the stage of differentiation of the cells or scenarioin force propagation through the ECM. These results show the role of magnetic mechano-conditioning in defining stem cell differentiation pathways and optimizing the temporal induction towards terminal osteogenesis utilizing combinations of chemical and mechanical conditioning. In addition these data provide useful insight into the modality of preconditioning hMSCs before transplantation.

## 3. Experimental Section

### 3.1. Cell Culture

Human bone marrow aspirates were purchased from Lonza (Slough, UK) and hMSCs were selected using the following plastic-adherent culture method. 0.5 mL of human bone marrow aspirate was added into T75 flask (Corning, Ewloe, UK) coated with 5 ng/mL fibronectin (Sigma, Dorset, UK) with 10 mL of proliferation medium [DMEM supplemented with 5% FBS, 0.5% antibiotics and 200 mM l-glutamine (all purchased from Lonza, Slough, UK)]. After 1 week of culture at 37 °C, half volume of medium was replaced with fresh medium and further cultured for another week. Several colonies were formed in each T75 flask and adherent cells were trypsinized for further expansion or were directly used in experiments. The osteogenesis of hMSCs was performed using the following methods. HMSCs were seeded in 6- or 24-well plates at the density of 10,000 cells/cm^2^ and allowed to adhere and proliferate overnight. The culture medium was replaced with osteogenic medium [α-MEM (Invitrogen) supplemented with 10% FBS (Lonza), 100 nM dexamethasone (Sigma), 50 μg/mL ascorbic acid and 10 mM β-glycerophosphate (BGP, Sigma)] on the next day. This medium was then changed every two days. Formation of mineralized nodules was determined on 6 or 24-well plates by alizarin red s histochemical staining [43].

### 3.2. Magnetic Particle Conjugation

Anti-human PDGFRα antibody (R & D Systems, Abingdon, UK) was conjugated to the surface of 250 nm superparamagnetic nanoparticles (Micromod, Rostock, Germany) after these particles had been functionalized with the Fc-specific secondary antibody (Sigma-Aldrich) according to the manufacturer’s protocol using 1-ethyl-3-(3-dimethylaminopropyl)-carbodiimide hydrochloride (EDAC) and *N*-hydroxysuccinimide (NHS) with the ratio of 20 μg of antibody per mg of MNP. For Arg-Gly-Asp (RGD, Sigma) conjugation, 80 μg of RGD was used for per mg of MNP (Micromode, 250 nm) following the similar protocol. The size and zeta potential of the MNPs with/without antibody conjugation was examined by A ZetaSizer (Malvern, Worcestershire, UK) at 25 °C, when dispersed in phosphate buffered saline (PBS) or in deionized H_2_O (dH_2_O).

### 3.3. Visualization of MNP Targeting Cell Membrane

For visualizing 250 nm particles targeting, cells were harvested using 4% paraformaldehyde after incubation with functionalized MNPs and removing unbound particles. Subsequently, cells were blocked for 30 min using 3% BSA. Then cells were incubated with anti-dextran antibody (STEMCELL Technologies, Sirocco, France) for 1 h at room temperature (RT) followed by FITC-conjugated secondary antibody (Santa Cruz, Heidelberg, Germany) for 1 h at RT. Subsequently cells were incubated either with Phalloidin-Atto 565 or with propidium iodide (PI) for 45 min at RT. MNPs targeting the cell membrane was assessed under confocal microscopy (Olympus, Southend-on-sea, UK).

### 3.4. Application of MFB Technique in Osteogenesis of hMSC

The MFB system (MICA Biosystems, Stoke-on-trent, UK) consists of horizontal arrays of NdFeB magnets, onto which cell culture plates can be situated (Figure 1). The frequency and amplitude of the oscillations of the array are controlled via a computerized stepper motor system. The field strength produced by the magnetic arrays of MFB in the vicinity of the cells is in the region of 60–120 mT with a field gradient of 3.3–11.0 Tm^−1^ [15]. In order to test the effects of mechanoreceptor stimulation via magnetic MFB exposure on hMSC mineralization, cells were incubated with antibody-conjugated MNPs for 1.5 h (25 μg particle suspension per well) after exposure to serum-free medium for 2 h. After washing with PBS twice to remove unbound MNPs, the MFB was used to apply translational force to the receptor- bound MNPs. All samples receiving MFB exposure treatment followed the same protocol unless specified. The cells were cultured with or without magneto-mechanical stimulation over indicated periods after which differentiation and mineralization was assessed using histology staining and FTIR, and mRNA expression was characterized using qRT-PCR.

### 3.5. Investigation of Temporal Effects of MFB on Osteogenesis of hMSCs

HMSCs were seeded onto 6-well plates and cultured overnight in proliferation medium. This was replaced with fresh osteogenic medium on the next day and cells were assigned into 2 groups—T1 and T2. The T1 group was pre-treated with osteogenic medium for 2 days, before exposing the cells to 5 days of magnetic conditioning using MFB for 1 h per day, targeting the mechanoresponsive receptor PDGFRα or integrin α_ν_β_3_. The T2 group was treated with magnetic conditioning directly after removing proliferation medium using the MFB at 1 h per day targeting either PDGFRα or integrin α_ν_β_3_ for 7 days. Two groups of cells were then harvested and the relative mRNA expression levels of Runx2, Col I, ALKP and OCN were measured by qRT-PCR.

### 3.6. qRT-PCR

Total mRNA was extracted by using TRI Reagent (Sigma, Dorset, UK) according to the manufacturer’s instructions. The quantity and quality of mRNA samples were checked using a NANODROP 2000 (Thermo, Wilmington, DE, USA). 1 μg of each mRNA sample was then reverse transcribed into cDNA using a QuantiTect Reverse Transcription Kit (Qiagen, Manchester, UK). Relative quantification by PCR was performed using Applied Biosystems reagents in 25 μL reaction systems containing 50 ng cDNA, 12.5 μL of 2× SYBR^®^ PCR Master Mix, 1 μL of Primer (Qiagen, Manchester, UK) and a STRATGENE Mx3005P QPCR system (Agilent, Wokingham, UK). The cycle conditions were set according to the manufacturer’s protocol. The ΔΔ*Ct* methodology was used to analyse gene expression and GAPDH was employed as the housekeeping gene. All results were normalized to the non-treated control group respectively.

### 3.7. Histology

#### 3.7.1. Alkaline Phosphatase and Von Kossa Double Staining

Cells were fixed with 4% paraformaldehyde for 1–2 min and then rinsed with TBST buffer [20 mM Tris-HCl (pH 7.4), 0.15 M NaCl, and 0.05% Tween-20] briefly to remove paraformaldehyde. The 500 μL of dye containing Fast Red Violet and Naphthol AS-BI Phosphate solution and deionized H_2_O (dH_2_O) in a ratio of 2:1:1 was added to each well and incubated at RT for 15 min in dark. Once ready, samples were thoroughly rinsed 4 times with dH_2_O. Freshly prepared 2.5% silver nitrate solution was administrated into each sample and incubated for 30 min in dark. After rinsing 4 times with dH_2_O, samples were incubated in 5% sodium carbonate in 25% formaldehyde for 5 min. Samples were rinsed 3 times with dH_2_O and ready for examination under light microscopy (Leica, Milton Keynes, UK).

#### 3.7.2. Calcium Staining

At the end of experiments, cells were fixed with 4% paraformaldehyde for 15 min and rinsed 3 times with PBS and 1 time with dH_2_O. 1 mL of freshly prepared 1% Alizarin Red S (Sigma, Dorset, UK) was administrated into each well and incubated at room temperature for 20 min. Stained samples were carefully rinsed 5 times with dH_2_O to remove physically trapped dye and examined under a Leica microscope.

#### 3.7.3. Collagen Staining

Collagen staining was conducted using a picro-sirius red method [44]. In short, cells were fixed with 10% neutralized formalin solution for 15 min and then washed 3 times with PBS. Nuclei were stained with haematoxylin (Sigma, Dorset, UK) for 8 min and then washed with running tap H_2_O. 500 μL of picro-sirius red solution [0.1% (*w*/*v*)], prepared in saturated aqueous picric acid (1.3%, *w*/*v*, Sigma), was administrated into each well containing fixed samples and incubated for 1 h. Afterwards, samples were rinsed with acidified H_2_O (5 mL of glacial acetic acid in 1 litre of dH_2_O) twice and examined under a Leica light microscope.

### 3.8. Thermal Gravimetric Analysis

To examine the pure mineral phase of some specimens, thermal gravimetric analysis (TGA) of the bone nodules were performed using a TGA instrument (SDT 600, TA Instruments, New Castle, DE, USA), which burnt out the organic components and impurities from samples. The TGA was set to a heating rate of 20 °C min^−1^ with heat cycles from 20 to 600 °C. The obtained mineral phase of specimens undergoing different culture conditions were further analyzed by FTIR.

### 3.9. FTIR

Investigation of the mineral composition of generated nodules was performed by using FTIR spectroscopy (Bruker Alpha P, Coventry, UK) equipped with a single reflection diamond attenuated total reflectance (ATR) sampling module. At the end of culture, samples were harvested and dried at 40 °C for 48 h. The FTIR spectra were obtained by recording scans between 4000 and 400 cm^−1^ with a resolution of 4 cm^−1^ with the FTIR spectrometer. Plots were baseline corrected and analyzed over the range of 500 to 1725 cm^−1^. After initial FTIR analysis, some samples were processed by TGA as described previously. The ashes that remained after the TGA procedure were collected from TGA sample holders and analyzed again by FTIR spectroscopy. Three spectroscopic parameters were calculated: mineral-to-matrix ratio, crystallinity, and collagen XLR.

### 3.10. Statistical Analysis

Data are presented as the mean ± standard error of the mean (SEM). Differences between groups were examined for statistical significance with one-way analysis of variance (ANOVA) using the Tukey least significance test for post-hoc comparisons. A minimal probability value of less than 5% was considered as statistically significant difference.

## 4. Conclusions

In this study, we have demonstrated that the osteogenesis of hMSCs is significantly affected by mechanical stimulation via magnetic tagging. By initially targeting the cell membrane receptor PDGFRα, we have demonstrated that a significantly higher mineral:matrix ratio was present in the cells after 3 weeks of magneto-mechanical stimulation combined with osteogenic medium culture. Moreover, we have revealed the temporal effects of mechanical stimulation associated with the kinetics of ostegenesis and mineralization. Both over a short term (7 days) and the medium term (12 days) of culture, osteogenic marker genes were expressed at higher levels in response to the 2 day-lag prior to mechanical stimulation compared with immediate mechanical stimulation, but with a different response profile. Over a 7-day culture, cells showed a strong response to PDGFRα-conjugated MNP treatment; whereas in the medium stage, cells showed a strong response to RGD-conjugated MNP treatment. Taken together, this study has explored effect of the temporal factor and targeting site, which are crucial to MFB technique applications in tissue engineering and has enriched the knowledge of mechanical stimulation for bone construct engineering.

## Figures and Tables

**Table 1:** The results of FTIR spectroscopy analyses, giving a comparison of the mineralization in magnetically stimulated [platelet-derived growth factor receptor α (PDGFRα) and RGD treatment] and non-treated (Cell) human bone marrow-derived mesenchymal stem cells (hMSCs) following 3 weeks of culture (*n* = 3, mean ± SD). Mineral crystallinity is a parameter that corresponds to the crystallite size and perfection, and was calculated from the intensity ratios of sub-bands at 1030 (stoichiometric apatite) and 1020 cm^−1^ (nonstoichiometric apatite) [33]. Collagen cross-link ratio (XLR) is a parameter reflecting the relative ratio of nonreducible and reducible collagen cross-links, expressed as the absorbance ratio at two specific wavenumbers (1660 and 1690 cm^−1^) [34].

Group	Mineral/matrix ratio	1660/1690 ratio	1020/1030 ratio
PDGFRα treatment	3.56 ± 0.52	2.48 ± 0.02	1.08 ± 0.01
RGD treatment	2.82 ± 0.56	2.45 ± 0.08	0.07 ± 0.01
Cell (nontreated)	2.53 ± 0.44	2.55 ± 0.04	1.06 ± 0.06

## References

[1] Langer R., Vacanti J.P. (1993). Tissue engineering. Science.

[2] Alsberg E., Feinstein E., Joy M.P., Prentiss M., Ingber D.E. (2006). Magnetically-guided self-assembly of fibrin matrices with ordered nano-scale structure for tissue engineering. Tissue Eng.

[3] Marolt D., Knezevic M., Vunjak-Novakovic G. (2010). Bone tissue engineering with human stem cells. Stem Cell Res. Ther.

[4] Baas E., Kuiper J.H., Yang Y., Wood M.A., El Haj A.J. (2010). *In vitro* bone growth responds to local mechanical strain in three-dimensional polymer scaffolds. J. Biomech.

[5] Cartmell S., El Haj A.J., Chaudhuri J.B., Rubeai M.A. (2005). Mechanical Bioreactors for Tissue Engineering. Bioreactors for Tissue Engineering, Principles, Design and Operation.

[6] Yamaguchi-Sekino S., Sekino M., Ueno S. (2011). Biological effects of electromagnetic fields and recently updated safety guidelines for strong static magnetic fields. Magn. Reson. Med. Sci.

[7] Kang K.S., Hong J.M., Kang J.A., Rhie J.W., Jeong Y.H., Cho D.W. (2013). Regulation of osteogenic differentiation of human adipose-derived stem cells by controlling electromagnetic field conditions. Exp. Mol. Med.

[8] Lewin M., Carlesso N., Tung C.H., Tang X.W., Cory D., Scadden D.T., Weissleder R. (2000). Tat peptide-derivatized magnetic nanoparticles allow *in vivo* tracking and recovery of progenitor cells. Nat. Biotechnol.

[9] Shimizu K., Ito A., Yoshida T., Yamada Y., Ueda M., Honda H. (2007). Bone tissue engineering with human mesenchymal stem cell sheets constructed using magnetite nanoparticles and magnetic force. J. Biomed. Mater. Res. B.

[10] Yamamoto Y., Ito A., Fujita H., Nagamori E., Kawabe Y., Kamihira M. (2011). Functional evaluation of artificial skeletal muscle tissue constructs fabricated by a magnetic force-based tissue engineering technique. Tissue Eng. Part A.

[11] Fayol D., Frasca G., Le Visage C., Gazeau F., Luciani N., Wilhelm C. (2013). Use of magnetic forces to promote stem cell aggregation during differentiation, and cartilage tissue modeling. Adv. Mater.

[12] Pankhurst Q.A., Thanh N.T.K., Jones S.K., Dobson J. (2009). Progress in applications of magnetic nanoparticles in biomedicine. J. Phys. D.

[13] Cartmell S., Dobson J., Verschueren S., El Haj A. (2002). Development of magnetic particle techniques for long-term culture of bone cells with intermittent mechanical activation. IEEE Trans. Nanobioscience.

[14] Hu B., Dobson J., El Haj A.J. (2013). Control of Smooth Muscle α-actin (SMA) up-regulation in HBMSCs using remote magnetic particle mechano-activation. Nanomedicine.

[15] Dobson J., Cartmell S.H., Keramane A., El Haj A.J. (2006). A magnetic force mechanical conditioning bioreactor for tissue engineering, stem cell conditioning and dynamic *in vitro* screening. IEEE Trans. Nanobioscience.

[16] Hughes S., McBain S., Dobson J., El Haj A.J. (2008). Selective activation of mechanosensitive ion channels using magnetic particles. J. R. Soc. Interface.

[17] Kanczler J.M., Sura H.S., Magnay J., Green D., Oreffo R.O., Dobson J.P., El Haj A.J. (2010). Controlled differentiation of human bone marrow stromal cells using magnetic nanoparticle technology. Tissue Eng. Part A.

[18] Thomas G.P., El Haj A.J. (1996). Bone marrow stromal cells are load responsive *in vitro*. Calcif. Tissue Int.

[19] Nii M., Lai J.H., Keeney M., Han L.H., Behn A., Imanbayev G., Yang F. (2013). The effects of interactive mechanical and biochemical niche signaling on osteogenic differentiation of adipose-derived stem cells using combinatorial hydrogels. Acta Biomater.

[20] Dobson J. (2008). Remote control of cellular behaviour with magnetic nanoparticles. Nat. Nanotechnol.

[21] Dobson J. (2010). Cancer therapy: A twist on tumour targeting. Nat. Mater.

[22] Chaudhary L.R., Hofmeister A.M., Hruska K.A. (2004). Differential growth factor control of bone formation through osteoprogenitor differentiation. Bone.

[23] Mitlak B.H., Finkelman R.D., Hill E.L., Li J., Martin B., Smith T., D’Andrea M., Antoniades H.N., Lynch S.E. (1996). The effect of systemically administered PDGF-BB on the rodent skeleton. J. Bone Miner. Res.

[24] Nash T.J., Howlett C.R., Martin C., Steele J., Johnson K.A., Hicklin D.J. (1994). Effect of platelet-derived growth factor on tibial osteotomies in rabbits. Bone.

[25] Vikjaer D., Blom S., Hjørting-Hansen E., Pinholt E.M. (1997). Effect of platelet-derived growth factor-BB on bone formation in calvarial defects: An experimental study in rabbits. Eur. J. Oral Sci.

[26] Ranly D.M., McMillan J., Keller T., Lohmann C.H., Meunch T., Cochran D.L., Schwartz Z., Boyan B.D. (2005). Platelet-derived growth factor inhibits demineralised bone matrix-induced intramuscular cartilage and bone formation. A study of immuno compromised mice. J. Bone Joint Surg. Am.

[27] Fierro F., Illmer T., Jing D., Schleyer E., Ehninger G., Boxberger S., Bornhäuser M. (2007). Inhibition of platelet-derived growth factor receptorbeta by imatinib mesylate suppresses proliferation and alters differentiation of human mesenchymal stem cells *in vitro*. Cell Prolif.

[28] Grey A., O’Sullivan S., Reid I.R., Browett P. (2006). Imatinib mesylate, increased bone formation, and secondary hyperparathyroidism. N. Engl. J. Med.

[29] O’Sullivan S., Naot D., Callon K., Porteous F., Horne A., Wattie D., Watson M., Cornish J., Browett P., Grey A. (2007). Imatinib promotes osteoblast differentiation by inhibiting PDGFR signaling and inhibits osteoclastogenesis by both direct and stromal cell-dependent mechanisms. J. Bone Miner. Res.

[30] Fitter S., Dewar A.L., Kostakis P., To L.B., Hughes T.P., Roberts M.M., Lynch K., Vernon-Roberts B., Zannettino A.C. (2008). Long-term imatinib therapy promotes bone formation in CML patients. Blood.

[31] Karp J.M., Ferreira L.S., Khademhosseini A., Kwon A.H., Yeh J., Langer R.S. (2006). Cultivation of human embryonic stem cells without the embryoid body step enhances osteogenesis *in vitro*. Stem Cells.

[32] Boskey A.L., Mendelsohn R. (2005). Infrared spectroscopic characterization of mineralized tissues. Vib. Spectrosc.

[33] Mendelsohn R., Paschalis E.P., Boskey A.L. (1999). Infrared spectroscopy, microscopy, and microscopic imaging of mineralizing tissues. Spectra-structure correlations from human iliac crest biopsies. J. Biomed. Opt.

[34] Paschalis E.P., Verdelis K., Mendelsohn R., Boskey A., Yamauchi M. (2001). Spectroscopic determination of collagen cross-links in bone. J. Bone Miner. Res.

[35] Komori T. (2002). Runx2, a multifunctional transcription factor in skeletal development. J. Cell. Biochem.

[36] Graham S., Leonidou A., Lester M., Heliotis M., Mantalaris A., Tsiridis E. (2009). Investigating the role of PDGF as a potential drug therapy in bone formation and fracture healing. Expert Opin. Investig. Drugs.

[37] Dobrowolski R., de Robertis E.M. (2011). Endocytic control of growth factor signalling: Multivesicular bodies as signalling organelles. Nat. Rev. Mol. Cell Biol.

[38] Kulterer B., Friedl G., Jandrositz A., Sanchez-Cabo F., Prokesch A., Paar C., Scheideler M., Windhager R., Preisegger K.-H., Trajanoski Z. (2007). Gene expression profiling of human mesenchymal stem cells derived from bone marrow during expansion and osteoblast differentiation. BMC Genomics.

[39] Golub E.E., Boesze-Battaglia K. (2007). The role of alkaline phosphatase in mineralization. Curr. Opin. Orthop.

[40] Kaazempur Mofrad M.R., Abdul-Rahim N.A., Karcher H., Mack P.J., Yap B., Kamm R.D. (2005). Exploring the molecular basis for mechanosensation, signal transduction, and cytoskeletal remodeling. Acta Biomater.

[41] Pedersen J.A., Swartz M.A. (2005). Mechanobiology in the third dimension. Ann. Biomed. Eng.

[42] Wang N., Butler J.P., Ingber D.E. (1993). Mechanotransduction across the cell surface and through the cytoskeleton. Science.

[43] Bodine P.V., Trailsmith M., Komm B.S. (1996). Development and characterization of a conditionally transformed adult human osteoblastic cell line. J. Bone Miner. Res.

[44] Junqueira L.C., Bignolas G., Brentani R.R. (1979). Picrosirius staining plus polarization microscopy, a specific method for collagen detection in tissue sections. Histochem. J.

